# Perforation of the Schneiderian membrane during sinus floor elevation: a risk factor for long-term success of dental implants?

**DOI:** 10.1007/s10006-020-00829-8

**Published:** 2020-01-30

**Authors:** Benedicta E. Beck-Broichsitter, Mirko Gerle, Jörg Wiltfang, Stephan Thomas Becker

**Affiliations:** 1 Department of Oral and Maxillofacial Surgery, Charité - University Medical Center Berlin, Augustenburger Platz 1, 13353 Berlin, Germany; 2 Department of Oral and Maxillofacial Surgery, Schleswig-Holstein University Hospital, Arnold-Heller-Straße 3, Haus 26, 24105 Kiel, Germany

**Keywords:** Sinus floor elevation, Surgical techniques, Perforation Schneiderian membrane, Complications

## Abstract

**Purpose:**

In cases of highly atrophic alveolar ridges, augmentation procedures became a frequent procedure to gain optimal conditions for dental implants. Especially in the maxilla sinus floor elevation procedures represent the gold standard pre-prosthetic and mainly successful procedure. The perforation of the Schneiderian is one of the most common complications. The aim of this study was to evaluate whether the intraoperative perforation of the Schneiderian membrane has an impact on long-term implant success.

**Methods:**

Thirty-four patients from a former study collective of the years 2005 and 2006 with a total of 41 perforations were invited for a follow-up examination to determine the long-term success rates after sinus floor elevation and subsequent implantation.

**Results:**

Twenty-one patients with 25 perforations were subsequently re-evaluated. One implant was lost due to a of periimplant infection after 232 days, resulting in an implant survival rate of 98% within a mean follow-up period of 8.9 years (± 1.5 years).

**Conclusion:**

Regarding the long-term success, there was no increased risk for implant failure or other persisting complications, e.g., sinusitis, after intraoperative perforation during sinus floor elevation in this study.

## Introduction

Since first reported in the 1980s, the sinus lift procedure has become the gold standard procedure for augmentation of the atrophic maxilla [1, 2], which is, besides common complications (e.g., bleeding, swelling), a highly predictive and successful procedure [3–5].

The perforation of the Schneiderian membrane represents one of the more specific complications during sinus floor elevation [6–9], occurring in a considerably wide incidental range from 10% up to 44% in the present literature [8, 10–19], whereas most studies set the incidence of perforations at a 20–25% among all sinus lift operations [20–22].

In many cases of intraoperative perforations, difficult anatomic circumstances were found, which might have contributed to a damage of the Schneiderian membrane. In a systematic review performed by Pommer et al., septa were found in 28.4% of intraoperative perforations [23]. Other reasons were summarized in other pathologic conditions (e.g., scaring) or very thin and vulnerable membranes [3].

Up to now, studies concerning complications during sinus lift procedures are further on underrepresented and the impact on long-term success is still not very well known. The aim of this study was to re-evaluate a cohort of 41 intraoperative perforations [24] of the Schneiderian membrane in 34 patients and its consecutive long-term influence on osseointegration, implant survival rates, and patients’ rehabilitation after dental implantation.

## Material and methods

### Patient recruitment

The study was conducted in accordance with the WMA Declaration of Helsinki - Ethical Principles for Medical Research Involving Human Subjects and was approved by the local Ethics committee (AZ 132/10). All patients were meticulously informed and gave their written consent for participation.

Two hundred one sinus floor elevations were counted in the Department of Oral and Maxillofacial Surgery at Schleswig-Holstein University Hospital of Kiel in the years between 2005 and 2006. In this collective of patients, the Schneiderian membrane was perforated during 41 procedures in 34 patients. For this retrospective evaluation, patient records were recollected and further assessed regarding implant insertions, implant failure, and further complications.

### Surgical procedure

Sinus elevation procedures were external sinus floor elevations through a bone window in the facial sinus wall in our study cohort. Augmentation of the sinus floor was performed with bone substitute material and bone filter material in defects less than 2 cm^3^, whereas above 2 cm^3^, whether mandible bone from the oblique ridge or iliac crest was harvested to augment the sinus floor in combination with bone substitute material. Dependent on size of the Schneiderian membrane perforation and in order to prevent sinusitis due to displaced graft material, membranes were whether sutured (Vicryl 6.0, Ethicon, Norderstedt, Germany) and covered with a resorbable membrane (BioGide, Geistlich, Wolhusen, Switzerland) in defects beyond of 5 mm in diameter. Smaller perforations were solely covered with a collagen membrane, fibrin glue, or left without any treatment. Sutures were removed 7 to 10 days after surgery. Due to the estimated primary stability, dental implants were inserted simultaneously or following a delayed implantation protocol.

### Medical record assessment

After implant insertion, yearly follow-up examinations were performed according to standardized protocols and panoramic radiographs were made 6 months after sinus floor elevation. Here, 21 patients regularly joined the routinely follow-up visits.

Patient records were screened for systemic diseases (e.g., diabetes mellitus), a previously diagnosed periodontitis or nicotine abuse.

Implant type, length and diameter were noted. Prosthodontic rehabilitation was distinguished in removable dentures and fixed bridgework. Origin of grafting material was further recorded. Simultaneously performed vertical augmentation was further noted as well as the position of the inserted implant. Implant success was defined as a prosthodontically integrated implant.

### Statistical assessment

Statistical data analyses was performed applying GraphPad Prism version 6.0 (GraphPad Software, La Jolla, CA, USA). Descriptive statistics were calculated and implant survival was displayed in a Kaplan-Meier plot. If the probability of error was less than 5%, the result was presented as statistically significant.

## Results

Among the 34 patients (24 female, 10 male) with 41 perforations of the Schneiderian membrane, a total of 25 perforations in 21 patients (13 female, 8 male) could be included in this retrospective long-term evaluation. Mean age of the patients was 63.8 years (SD 14.7 years) in the study cohort. The mean follow-up period in this study was 8.9 years (SD 1.5 years). Among the study groups, a total of 3 patients had diabetes type II (14.3%). Periodontitis was previously diagnosed in 13 patients (61.9%) and 13 patients were smokers (61.9%).

**Fig. 1:**
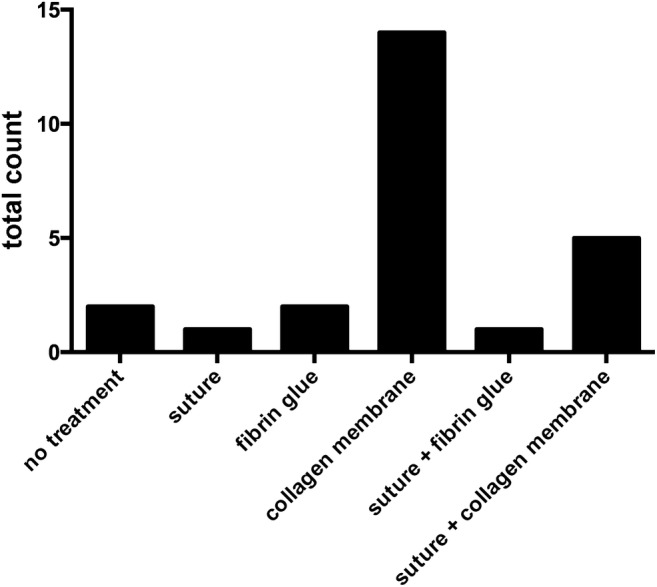
Surgical management of perforations

Perforation of the Schneiderian membrane was covered with a collagen membrane in 14 procedures (56%) and in 5 patients (20%), a suture was combined with a membrane. Figure 1 depicts the distribution of perforation management. In the study group, a total 49 implants were inserted, whereas 20 implants were inserted simultaneously to sinus floor augmentation and 29 implants in a second procedure (59.2%). Twenty-five implants inserted in the premolar region and 24 implants inserted in the molar region (Table 1).

**Table 1:** Distribution of implant position in the study cohort

	Premolar region		Molar region	
Implant position	04	05	06	07
Absolut count	11	14	22	2
Relative	22.4%	28.6%	44.9%	4.1%

**Fig. 2:**
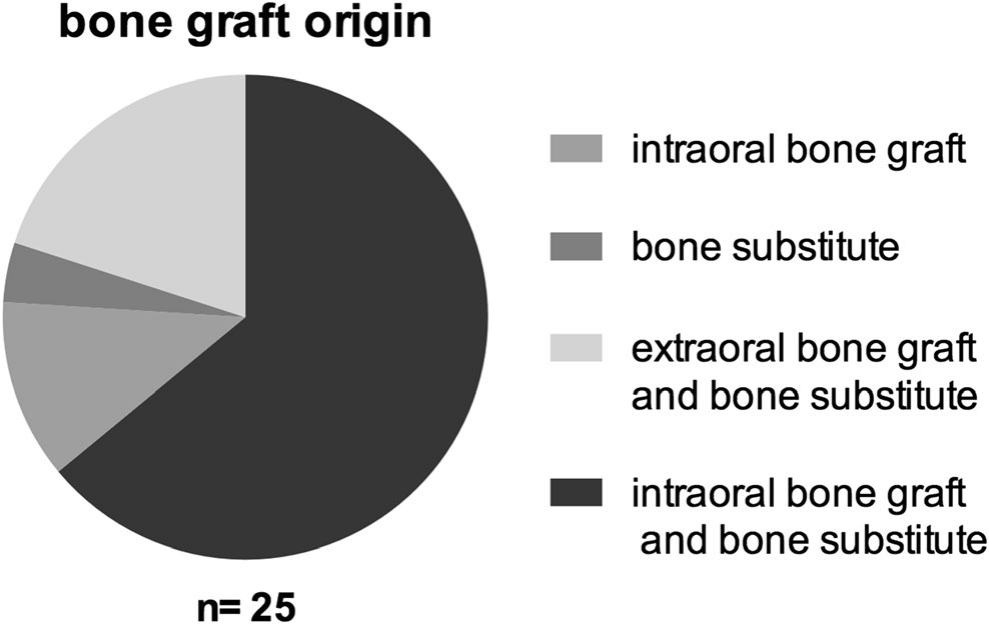
Bone graft origin during sinus floor elevation

Material for augmentation was mainly a mixture of intraorally harvested bone with bone replacement material in 16 procedures (64%) followed by a mixture of iliac crest and bone replacement material in 5 sinus lifting procedures (20%) (Fig. 2). One patient was treated with bone replacement material alone (4%) and 3 patients received an autologous bone graft originating from the mandible (14.3%). In 23.8% of cases (5 patients), an additional onlay grafting procedure was performed.

Six different implant types were used. Technical data and implant manufacturers are displayed in Tables 2 and 3. Twelve implants served prosthodontically in removable dentures (24.5%), whereas the remaining 37 implants were crowns and bridgework.

**Table 2:** Distribution of inserted implant types (manufacturers)

Implant type	Camlog	Straumann	Dentsply Frialit	Astra	Nobel Biocare	Ankylos
Absolut count	15	20	6	3	2	3

**Table 3:** Distribution of inserted implant sizes

Implant diameter	Implant length	Absolute count
3.3	12	2
3.5	11	2
3.8	13	1
4	13	2
4.1	10	1
4.1	12	10
4.1	14	7
4.3	11	6
4.3	13	8
4.3	16	2
4.3	15	3
4.5	13	3
4.5	11	1
4.5	15	1

**Fig. 3:**
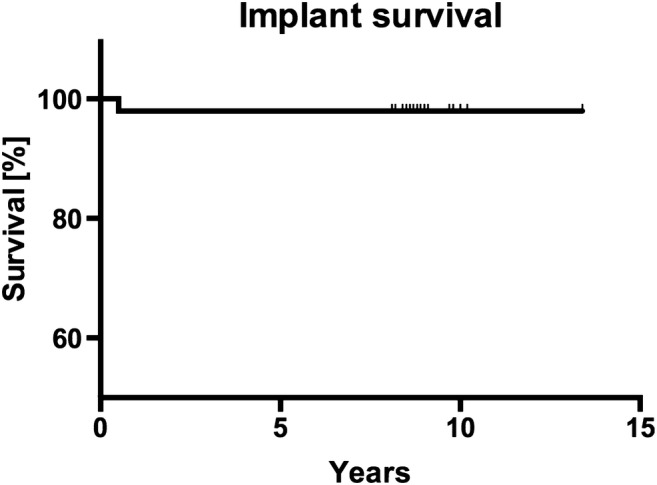
Kaplan-Meier curve of implant survival rate

Regarding short-term complication, one patient developed a dehiscence of the gingival wound leading to a delayed wound healing without any dissemination of infection. Another patient struggled with signs of sinusitis as previously reported [24]. The long-term evaluation revealed that among the 49 inserted implants, one was lost due to a periimplant infection after 232 days leading to an implant survival of 98% in Kaplan-Meier analysis (Fig. 3).

## Discussion

The aim of this re-evaluation of intraoperative perforations of the Schneiderian membrane in a cohort of previously reported 34 patients was to determine its long-term influence on the implants’ osseointegration, the survival rates, and patients’ rehabilitation after dental implantation [24]. Twenty-one patients with 24 perforations could be included in this approximately 9-year follow-up.

Perforating the Schneiderian membrane during sinus lifting procedure is mostly common and of vary between 10 and 44% [8, 10–19].

Alongside with the surgical experience of the surgeon, scared tissue due to previously performed surgery or infection may increase the risk to perforate the Schneiderian membrane [15, 25–27]. Variations of the anatomy including the shape of the lateral maxillary sinus wall, sinus septa, and the thickness of the membrane itself may represent further influcence factors for intraoperative perforations [15, 23, 25–27]. In our former study, collective thin membranes were observed in 27% of patients. Sinus septa were seen in 22% of the patients [24]. Other studies suggested that sinus floor elevation might be relatively contraindicated in anatomical variations like septa or mucosal swelling [16].

In the present study, one implant was removed due to early onset of periimplantitis in the first year after implantation. The resulting implant survival of 98% in our study after 8.9 years is in the comparable upper range of currently published studies with survival rates of 88 to 100% 10–14 years after dental implant insertion [28–33]. Another study evaluating the implant success over a 1-year period and the influence of the bone grafts’ origin on the complication rate did not reveal any significance regarding the occurrence of postoperative complications. Furthermore, no implant was lost during the observation period [19] and another study also did not reveal any connection between complications and membrane perforation during the observation period of 8 years [25]. In contrast to these studies, Nolan et al. reported about a statistically significant higher use of antibiotic treatment of sinus infections and higher rates of graft failure in 359 sinus floor elevation procedures with a reported perforation rate of 48.8% (Nolan et al. 2014).

The occurrence of complications might also be dependent on the surgical management in case of a due to the efficiency of perforation’s closure. So far, there are no existing guidelines to standardize perforation closure on an evidence-based level. The published studies suggest covering small perforations with collagen or demineralized laminar bone membranes or fibrin glue and an additional resorbable suture of larger perforations in order to close the dehiscence completely [8, 16, 22]. Other studies covered even larger perforations with membranes only and did not report of severe adverse events with clinical relevance [19, 21, 22, 34]. In our study, a perforation was not associated with a reduced implant success, but was in contrary found to result in a reduced implant survival rate in a study performed by Proussaefs et al. in 2004, when a perforation exceeded 2 mm. In another study, the implant survival rates after membrane reconstruction were found to inversely correlate with the size of the perforations [35].

In all these cases, a lateral approach is necessary in order to recognize and subsequently manage the perforation. In original cohort of the study, four procedures were terminated because of the extent of the perforation in two cases, vulnerable mucosa and a retention cyst in one case each. The procedure was performed again after 6 months without further complication [24]. This in accordance with other studies reporting disrupting surgery when the size of perforations are too extensive to repair [10, 20].

Besides the mode of membrane repair, the timing of implant placement might also influence the overall implant survival after membrane perforations. In our study, immediate implant was only placed immediately when a high primary stability could be ensured due to a good residual bone quality, which was in accordance with another study as well [36]. However, it was suggested that patients with a residual bone height between 1 and 3 mm were at risk for implant failures in a one-stage surgery with a lateral approach in sinus floor elevation [26]. In all other cases, the implants were inserted in a two-stage approach although the risk of complications might be increased. A previously published study, regardless of perforations, found that there was a significantly higher risk to develop a soft-tissue complication in the periimplant region due to a second procedure. Bone grafting was slightly not correlated with these complications but might implicate a potential influence as well [37].

In this context, other risk factors for implant success have to be considered, too. For example, the use of tobacco was found to have a negative impact on implant success after sinus floor elevation [38], when a total of 60 patients with 228 inserted implants were evaluated. Among smoking patients, implants success revealed 65.3% and therefore significantly lower compared to non-smokers with a success rate of 82.7%. Although some studies did not reveal a significant connection between the use of tobacco and implant survival [39, 40], it is widely accepted that smoking might represent a risk factor for wound healing disturbances and implant failure in several other studies [28, 41].

## Conclusion

Our results indicate that the perforation of the Schneiderian membrane had no long-term impact on implant success rates or persisting long-term complications if the surgical management allows to cover the perforation or to change the surgical protocol to a two-stage surgery.
